# A143 PEDIATRIC EOSINOPHILIC ESOPHAGITIS IN CANADA: A MULTI-CENTER COHORT WITH FOCUS ON THE STRICTURING PHENOTYPE

**DOI:** 10.1093/jcag/gwac036.143

**Published:** 2023-03-07

**Authors:** D Burnett, V Avinashi, T Hoang, A Otley, R Persad, M Sherlock, H Huynh

**Affiliations:** 1 Dalhousie University, Halifax; 2 University of Saskatchewan, Saskatoon; 3 University of British Columbia, Vancouver; 4 University of Alberta, Edmonton; 5 McMaster University, Hamilton, Canada

## Abstract

**Background:**

Eosinophilic esophagitis (EoE) is a chronic eosinophil-predominant esophageal inflammatory condition, and is now recognized as one of the most common organic causes of dysphagia in pediatrics. While fibrostenotic esophageal strictures are a common complication of adult EoE, characterization of the stricturing phenotype in pediatric EoE remains at an early stage.

**Purpose:**

Describe the Canadian pediatric EoE experience, with focus on the stricturing phenotype.

**Method:**

New pediatric EoE diagnoses from 2015-2018 were retrospectively identified in Vancouver (BC), Northern Alberta (AB), Hamilton (ON) and Nova Scotia (NS). Incidence rates were calculated using 2016 Federal census data. Clinical, endoscopic and histologic data were gathered for each patient’s initial clinical encounter and for any esophagogastroduodenoscopies (EGD) until the end of the follow-up period (fall 2019).

**Result(s):**

332 new EoE cases were identified during the study period across all 4 sites. The incidence of EoE in patients less than 15 years old was 9.1 (AB), 6.5 (NS), 3.1 (BC) and 5.4 (combined) per 100,000 person-years. Incidence was not calculated for Hamilton due to risk of ascertainment bias given their catchment area overlaps with multiple other centers. Of identified cases, 40 (12.0%) had endoscopically-identified esophageal strictures at diagnosis or during the follow-up period, with a subset of 11 (3.3%) of these patients undergoing mechanical esophageal dilation. Another 11 (3.3%) patients had more subtle signs of esophageal narrowing (ex. mucosal rents), while the majority had no evidence of esophageal narrowing (281, 84.6%). The median age at diagnosis was higher in the cohort with strictures (12.4 years, IQR 8.9-14.1) than those without (10.3 years, IQR 6.1-13.6) but with wide IQR's. A similar trend was seen for the median duration of symptoms at diagnosis (1.5 years, IQR 0.5-3 versus 1.0 years, IQR 0.6-2.8). Patient reported food bolus impaction (OR 9.8, 95% CI 3.9-19.9) and dysphagia (OR 3.3, 95% CI 1.3-7.8) were associated with stricturing disease, with other symptoms less clearly linked. Trachealization was the endoscopic finding most closely associated with esophageal strictures (OR 5.7, 95% CI 2.8-11.5). Esophageal stricture(s) were noted on 72 EGDs, including 27 endoscopic esophageal dilations, but excluding 10 esophageal dilations by interventional radiology. Of the 65 EGDs done in follow-up of a known esophageal stricture (see Table), 4 of 31 had resolution of this finding post mechanical dilation, and 19 of 39 had resolution of the stricture after initiation of new medical or dietary treatments (without dilation).

**Conclusion(s):**

EoE is common in Canadian children, with esophageal strictures within a few years of diagnosis in 12% of cases. Interestingly, a large portion of strictures resolved without mechanical dilation, which seems to point away from fibrosis as the primary driver of esophageal strictures in this patient population.

**Please acknowledge all funding agencies by checking the applicable boxes below:**

CAG, Other

**Please indicate your source of funding;:**

Women and Children's Health Research Institute

**Disclosure of Interest:**

None Declared

